# Aqueous Extract of Cinnamon (*Cinnamomum* spp.): Role in Cancer and Inflammation

**DOI:** 10.1155/2023/5467342

**Published:** 2023-05-11

**Authors:** Shubrata Khedkar, Minhaj Ahmad Khan

**Affiliations:** Department of Biochemistry, Lovely Professional University, Jalandhar 144411, Punjab, India

## Abstract

Cinnamon (*Cinnamomum* spp.; family Lauraceae), a plant widely used as a spice and flavoring agent and in the perfume industry, has high therapeutic value. However, the components and chemical properties of cinnamon extracts vary depending on the part of the plant, the method, and the solvent used for extraction. Green extraction methods using safe and green solvents have gained increased interest in recent years. Water is an environmentally friendly and safe green solvent widely used for preparing cinnamon extracts. This review focuses on the various preparation techniques for the aqueous extract of cinnamon, its major bioactive components, and their beneficial roles in different pathological conditions, specifically cancer and inflammation. The aqueous extract of cinnamon contains several bioactive compounds, such as cinnamaldehyde, cinnamic acid, and polyphenols, and exerts anticancer and anti-inflammatory properties by altering key apoptotic and angiogenic factors. The whole extract is a better anticancer and anti-inflammatory agent than the purified fractions, indicating a synergistic effect between various components. Studies have indicated that aqueous cinnamon extract has immense therapeutic potential, and to better understand its synergistic effects, extensive characterization of the aqueous extract and its potential to be used with other therapies should be explored.

## 1. Introduction

**Figure 1:**
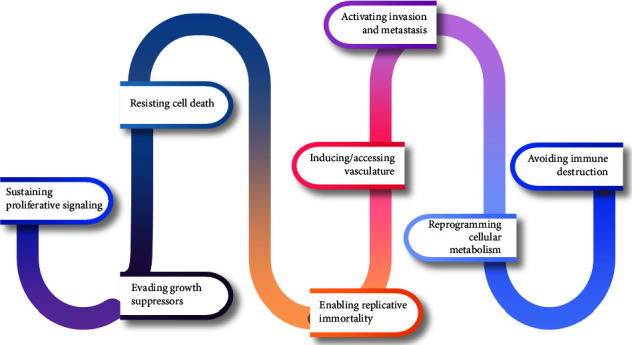
Hallmark of cancer cells.

According to 2020 statistics, cancer accounts for approximately 10 million cancer-related deaths with approximately 19.3 million cancer cases worldwide [1]. Carcinogenesis is the progressive transformation of normal cells into neoplastic cells, comprising four phases: tumor initiation, tumor promotion, malignant conversion, and tumor progression. The first phase of carcinogenesis, tumor initiation, is marked by changes in DNA (deoxyribonucleic acid) brought about by physical or chemical carcinogenic stimuli [2]. Changes in DNA lead to oncogene-mediated activation or tumor suppressor gene-mediated suppression of various genes [2, 3]. During tumor promotion, genes involved in cell proliferation and vascularization are activated, resulting in the formation of neoplastic cells. In the malignant conversion step, the preneoplastic cells transform into cells expressing malignant phenotypes. In the tumor progression stage, the tumor aggressively proliferates and invades other tissues, accumulating several different mutations [3]. Hanahan [4] reviewed the hallmarks of cancer, providing a detailed description of changes occurring in a cell moving from a normal to a transformed state (Figure 1).

Approximately 25% of cancer cases have a history of chronic inflammation or infectious diseases [5, 6]. Extrinsic factors, including smoking and infections, and intrinsic factors, such as tumor-associated inflammation, contribute to an inflammatory tumor microenvironment (TME) [7]. Additionally, changes in cell physiology and metabolism result in an increase in stress-related inflammatory markers [8]. Cancer treatment using the conventional approach and biotherapeutics could also induce inflammation and has been extensively studied in recent years [6].

Inflammation is a protective response elicited by cells when tissue damage is caused by infection, trauma, exposure to a toxin, or physical or ischemic injury. A successful inflammatory response leads to homeostasis and inflammation resolution upon the removal of the stimulus [7, 9]. However, a standoff between the immune response and a stimulus that cannot be eradicated results in chronic inflammatory conditions [6, 10]. In chronic inflammatory conditions, inflammatory mediators such as reactive oxygen species cause DNA damage by oxidation and facilitate spontaneous mutagenesis mediated by 8-oxo-7-hydro-2′-deoxyguanosine [11]. The interplay between various proinflammatory factors in the TME plays a significant role in cancer development and progression [12, 13]. Therefore, considerable research has been conducted in the last decade to understand the relationship between inflammation and cancer [7, 14, 15].

One of the most well-studied genes involved in tumor initiation and progression is the mutated tumor suppressor gene *Tp53*, encoding the p53 protein that plays a pivotal role in cell homeostasis [16]. Wild-type p53 suppresses tumor growth by activating factors responsible for DNA repair and inducing apoptosis and senescence [16], prevents oncogenic transformation in cultured cells, and p53 null mice are highly likely to develop tumors [17]. Recent studies have reported the role of mutant p53 in the TME. Bhatta et al. [18] have reported that the presence of mutant p53 protein in the TME of nontransformed cells promotes tumor progression through cell reprogramming. The cross-talk between the TME and the tumor is enhanced by mutant p53, resulting in the increased transcription of inflammatory messengers such as nuclear factor kappa B (NF*κ*B) [19]. A mutated p53 protein results in unchecked NF-kB activity, causing increased expression of proinflammatory signaling molecules; the resulting TME is characterized by hypoxia and increased ROS (reactive oxygen species) activity, further activating redox-sensitive factors such as hypoxia-inducible factor (HIF-1*α*) [6, 20]. Hypoxia also increases the levels of proinflammatory cytokines, including interleukin 6 (IL-6), tumor necrosis factor (TNF-*α*), and proangiogenic factors, including the vascular endothelial growth factor (VEGF), resulting in chronic inflammation [6, 20]. An interplay of factors, p53, along with increased IL-6, results in increased activity of a signal transducer and activator of transcription (STAT-3), supporting tumor induction and providing a favorable environment for tumor growth [21]. Therefore, p53, IL6, VEGF, and STAT3 are attractive therapeutic targets for cancer treatment [22].

Complementary and alternative medicine (CAM) is gaining more attention among patients with cancer. Approximately 87% of the patients use at least one form of CAM, including herbal medicine, yoga, reiki, and naturopathy, to improve their overall quality of life [23]. Furthermore, ∼22% of patients are reported to opt for herbal medicines along with conventional therapies [24]. Plant polyphenols are potent anticancer and anti-inflammatory agents and have been reported to reduce the side effects caused by chemotherapy and reverse prolonged treatment-induced chemoresistance [25]. Even though several natural compounds such as curcumin, resveratrol, green tea extracts, quercetin, and lycopene have been used for clinical testing, the mechanism of action of these natural compounds and how they exert their effects remain largely unidentified [26]. Several widely used spices, such as curcumin (from turmeric, *Curcuma longa*) [27, 28], eugenol (clove, *Syzygium aromaticum*) [29], cinnamic acid, and trans-cinnamaldehyde (*Cinnamomum* spp.) [30], have been identified to have anticancer, anti-inflammatory, and antioxidative properties [31] and are the source of many active therapeutic compounds. This review focuses on cinnamon and details its extraction techniques, active compounds, and their therapeutic potentials.

## 2. Cinnamon and Extraction Methods

Cinnamon (*Cinnamomum* spp.) is a tropical plant belonging to the Lauraceae family and has been widely used as a spice, flavoring agent, and in the perfume industry since ancient times. *Cinnamomum* comprises ∼250 species, of which true cinnamon or Ceylon cinnamon (*Cinnamomum verum*), Cassia cinnamon or Chinese cinnamon (*Cinnamomum cassia*), Indonesian cassia (*Cinnamomum burmannii*), and Vietnamese cinnamon (*Cinnamomum loureiroi*) are most commonly used [32]. Increasing research on this plant has unraveled several benefits of cinnamon consumption. Various parts of the cinnamon plant, such as the bark, leaves, flowers, and fruits, are used to extract the cinnamon essential oil with different active compounds such as cinnamaldehyde, eugenol, camphor, and trans-cinnamyl acetate, respectively [33], and therapeutic properties (such as anti-inflammatory, antidiabetic, antioxidant and antimicrobial). Apart from essential oils, the anti-inflammatory and anticancer therapeutic potentials of organic and aqueous extracts of cinnamon have also been reported [29, 30], and several extraction methods using various solvents have been developed over the years [34–36]. The aqueous polyphenol fraction has therapeutic potential as an anti-inflammatory agent for the treatment of periodontal disease [37]. In addition, several reports have confirmed the therapeutic potential of the aqueous cinnamon extract against cancer [9, 38, 39]. The extraction method and type of solvent affect the recovery of polyphenols and, thus, the therapeutic properties of the cinnamon extract [40]. Abd Wahab and Adzmi [41] compared two types of cinnamon extract, Soxhlet extract using methanol and water extract. They recorded the cytotoxic effects of these extracts and compared the results based on the observed half maximal inhibitory concentration (IC50) values. They observed that the Soxhlet extract works better at the 24-hour time point (in an MTT (tetrazolium salt (3-(4,5-dimethylthiazol-2-yl)-2,5-diphenyltetrazolium bromide) assay) and is more effective than water extract. However, their lab observed higher IC50 for the extract than the published literature. They speculated that this difference might result from the difference in extract yield (∼21% in reported literature vs. 7% in their lab) and cinnamaldehyde concentration per gram. They also opined that cinnamaldehyde which is responsible for apoptosis and cell death in cancer cells affects the IC50 values.

In another example, an aqueous solution of ethanol (30–60%) was effective for phenol recovery and increasing the aqueous fraction resulted in better recovery of polyphenols [42]. An *in vivo* study showed no significant antidiabetic activity of the aqueous cinnamon extract in patients with type 2 diabetes. The overall polyphenol content of the extract was lower than what is reported in the literature, which could be attributed to the age of the tree, tree section, and extraction method used [43]. The impact of environmental factors, propagation method, age, and the plant's physical state (flowering, etc.) on phytochemicals has been reported [44]. With the seeding type of propagation (Sri Gamunu), a plant grown in a wet climate that is 3 -5 years old and harvested during flowering time contains a high phytochemical content. While with the vegetative type of propagation (Sri Wijaya), > 5-year-old bark had a greater yield of phytochemicals [44]. A study conducted on two accessions of C. zeylanicum, namely, Sri Gamunu and Sri Vijaya, revealed a difference in the composition of the bark oil. 78% of Sri Gamunu bark oil was cinnamyl acetate and cinnamaldehyde, while only 63% of Sri Vijaya contained these active ingredients. Sri Vijaya had higher levels of benzyl benzoate and eugenol [45]. Processing of plants affects the total phenol content of the spice. Klejdus and Kováčik [46] reported that complete tissue disruption is necessary for maximum recovery of total phenols. In their study, when whole bark was used, the recovery of total phenols was much less when compared to processed samples such as crushed materials using pestle and mortar, powdered using a grinder, or a combination of the two. Drying the cinnamon parts (leaves, bark, roots, etc.) is one of the critical postharvest processes. Reports indicate that drying impacts the phytochemical properties of cinnamon. A study conducted by Bernard et al. [47] showed that sun-drying was very destructive and resulted in the degradation of phytochemicals and, thus, the radicals scavenging activity when compared with a fresh sample. Oven drying was reported to be one of the better methods for preserving active ingredients. These studies indicate that researchers should be mindful when acquiring the samples and collect as much data about them as possible. Reporting the details is equally important so that others can truly benefit from the information. Ribeiro–Santos et al. [32] have written an excellent review that contains a section on plant processing and the effects it can have on the final extract.

**Figure 2:**
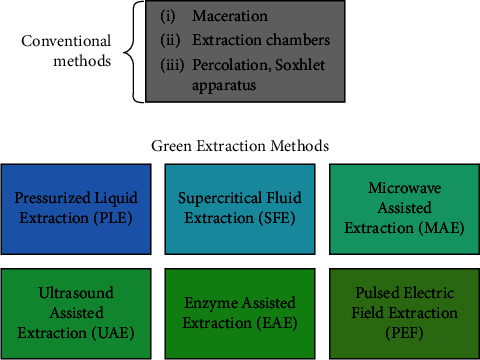
Conventional and green extraction methods.

Conventional methods such as maceration, extraction chambers, and percolation use organic solvents such as methanol and acetone for preparing plant extracts. However, these methods have a few drawbacks, they are not environmentally friendly, and it is crucial to ensure the removal of residual solvent from the final preparation before testing its effects [39]. Therefore, many researchers now prefer the green extraction method, which aims to discover innovative extraction processes that are safe, yield high-quality extracts, and reduce energy consumption [48]. One of the principles of green extraction is using alternate solvents, principally water and agrosolvents also known as biosolvents as they are produced from biological materials (for example, methyl esters of fatty acids in vegetable oil, ethanol from fermentation of sugar beet, and glycerol from vegetable oil transesterification), to minimize the release of toxic waste into the environment. Some of the green extraction methods (Figure 2) and their advantages and limitations are captured in Table 1. Green extraction techniques use modern technologies and sometimes also referred to as technology-assisted extraction methods [52]. It should be noted that one technique may not suffice the extract requirements. A combination of various technology-assisted green extraction methods can be used to achieve maximum yield of desired compounds [49, 52].

Subcritical water extraction, one of the green methods being performed at high temperatures (200°C) and pressure (1.5 MPa), has gained considerable attention [48, 53]. During subcritical extraction, the physicochemical properties of water, such as its dielectric constant, are significantly reduced (similar to ethanol), making it a suitable solvent for extracting low-polarity compounds [48]. Hydrodistillation is conventionally used to extract essential oils from cinnamon, and recent advances in this process include microwave-assisted hydrodistillation, which is energy-saving and environmentally friendly [52, 54]. Aqueous cinnamon extract preparation by boiling, preparing a decoction, and either lyophilizing or using it as such after sterile filtration have also been reported [55, 56].

Aqueous cinnamon extract has been extensively used in studies related to type 2 diabetes mellitus to manage blood sugar levels and as an antioxidant [57–59]. Comprehensive information on the role of cinnamon in diabetes management has been described in a recent review article [60]. In addition, cinnamon also acts as an antioxidant, anti-inflammatory, anticancer, and wound healing agent [30]. Cinnamon aqueous extract has been used as an anti-inflammatory agent in hepatorenal toxicity [61] and as an antiproliferative agent in human prostate cancer cells [62]. It has also been shown to inhibit tumor angiogenesis and growth [63]. As water is considered the most suitable form of solvent in terms of safety [64], we searched for references that would provide information on preparing aqueous cinnamon extracts with or without sophisticated instruments.

We searched various databases such as PubMed/MEDLINE, Scopus, Web of Science, Embase, and Google Scholar with keywords such as cinnamon aqueous extract, water extract of cinnamon, cinnamon and inflammation, cinnamon and cancer, aqueous extract and inflammation, and aqueous extract and cancer. Based on the literature search, we identified the need for a comprehensive review of aqueous extract preparation methods and their potential therapeutic properties. Therefore, this review aimed to highlight the role of the aqueous extract of cinnamon as a potential anticancer and inflammatory agent. To this end, we have summarized the published methods for aqueous extract preparations and their beneficial effects on cancer and inflammatory conditions.

## 3. Aqueous Extract of Cinnamon

### 3.1. Extraction Methods

**Figure 3:**
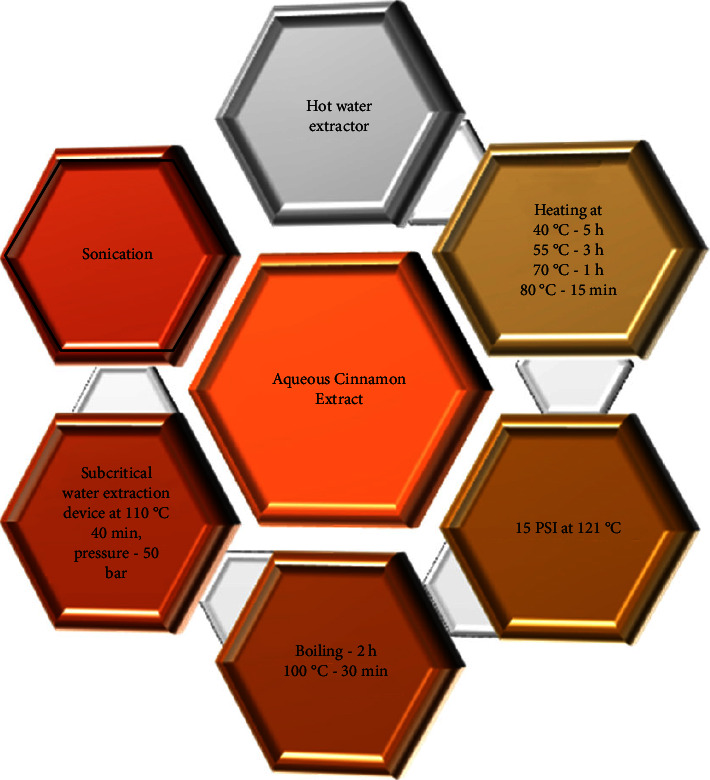
Summary of the methods used for preparation of aqueous cinnamon extract. Increased temperatures are used for extraction, but there is a lot of variation with respect to heating temperature and duration as seen in the figure. Similarly, even when boiling is used to prepare the extract, lab-specific methods have been used.

This review identified literature on relatively simple aqueous extraction methods that could be carried out in laboratories with limited resources (Figure 3 and Table 2). However, the literature search revealed that even seemingly simple extraction methods involve several steps, including extracting at 40°C, stirring rapidly for 10 min, centrifugation, chilling the beaker with the extract in an ice bath, and stirring, followed by filtration and lyophilization [55], to a simple one-step extraction process, where the ground cinnamon powder was resuspended in sterile water (70°C for 1 h), centrifuged, and used after filtration [67]. A common feature of most extraction methods summarized in Table 2 is that the extract was lyophilized and stored at −70°C to −80°C [66, 69, 70]. In most of the studies, ground/pulverized cinnamon was used [55, 65], while in some cases, the extract was prepared after soaking the bark in water [69].

### 3.2. Role of Aqueous Cinnamon Extract

**Figure 4:**
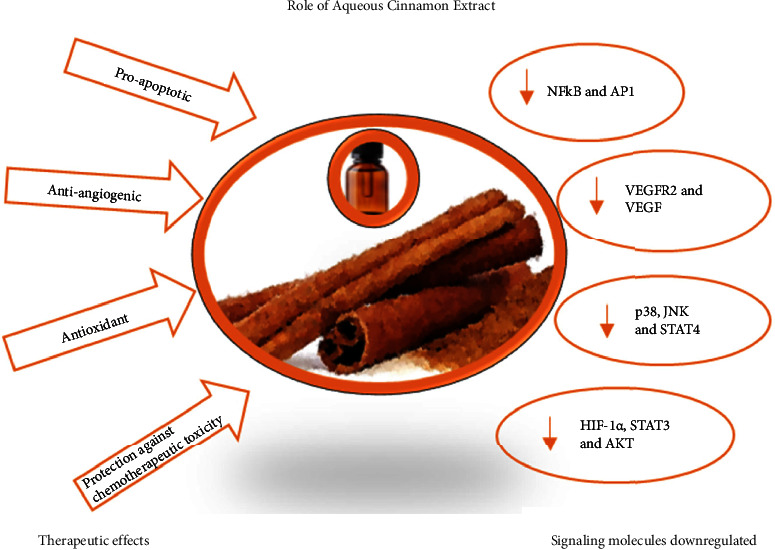
Aqueous cinnamon extract has been shown to have anticancer and anti-inflammatory properties. This figure depicts a simplistic view of the molecules downregulated in the process of counteracting cancer and inflammation by aqueous cinnamon extract. NF-kB: nuclear factor kappa light chain enhancer of activated B-cells, AP1: activator protein 1, VEGFR2: vascular endothelial growth factor receptor 2, VEGF: vascular endothelial growth factor, p38-MAPK: p38 mitogen-activated protein kinases, STAT4: signal transducer and activator of transcription 4, HIF-1*α*: hypoxia-inducible factor 1-alpha, STAT3: signal transducer and activator of transcription 3, and AKT: it refers to protein kinase B (PKB).

Aqueous cinnamon extract has been reported to have anticancer [65, 66], anti-inflammatory [37, 69], and antioxidant [71] properties and exert these effects by various mechanisms, a few of which are described in this section and summarized in Table 2 and Figure 4.

### 3.3. Anticancer Activity

Cinnamon extract is toxic to cancerous cells and inhibits tumor cell growth *in vitro* [41, 66, 72]. The cytotoxic effect of crude cinnamon aqueous extract was reported to be more potent than that of individual components such as cinnamaldehyde [66]. In an *in vitro* study, it was shown that aqueous cinnamon extract was toxic for oral squamous carcinoma cells (OSCC), reaching a 90% cytotoxicity with 10 mg/mL extract within 48 h. Moreover, treating OSCC with aqueous saffron and cinnamon extracts had a synergistic effect, with more cytotoxicity at lower concentrations [9].

Tumor cells require an abundant supply of nutrients and the removal of cellular metabolic waste for survival. The genes involved in angiogenesis (formation of new blood vessels) or proangiogenesis are upregulated in the TME [80]. Aqueous cinnamon extract has been shown to downregulate the proangiogenic factors [65] and exhibit antiangiogenic properties [63, 65, 67]. It exerts its effects at various stages of angiogenesis [67] and modulates the expression of VEGF mediated by the inhibition of cyclooxygenase (Cox-2) in an *in vitro* as well as *in vivo* system [65] and that of HIF-1*α*, STAT3, and protein kinase B (AKT) in an *in vitro* system [63]. Cinnamaldehyde [63] and procyanidin [67] were identified as active components in aqueous cinnamon extract, which inhibit VEGF expression and VEGFR2 signaling *in vitro*, respectively. An effective way to eliminate cancer cells is cytolysis which is mediated by CD8+ T cells. TME redesigns itself to evade the immune machinery and thus is refractory to the cytolytic CD8+ T cells [65]. Kwon et al. [65] have reported (*ex vivo* study) that cinnamon extracts enhance the cytolytic activities of CD8+ T cells which are associated with increased expression of perforin and granzymes.

NF*κ*B regulates many factors that play a role in cell multiplication and cell survival. Increase in NF*κ*B results in an increase in antiapoptotic molecules. One of the mechanisms by which tumor cells increase their chances of survival is by evading apoptosis; the mechanisms can vary from an increase in antiapoptotic molecules such as B-cell lymphoma 2 (BCL-2) and Bcl-2-like protein 4 (Bax), downregulation of death receptors, or inactivation of caspase-8 [81]. Many plant extracts, including aqueous cinnamon extract, act as proapoptotic agents [81]. One of the reported effects of the aqueous cinnamon extract both in *in vitro* and *in vivo* system was that it effectively inhibited the expression of proapoptotic genes, such as Bcl-2, BcL-xL, and survivin, which was mediated via a decrease in NFkB and activator protein AP-1 signaling [56]. Varalakshmi et al. [82] based on molecular docking studies have reported that procyanidin B2 has an inhibitory effect on NFkB and may act by inhibiting the translocation of this factor to the nucleus. Another mechanism by which aqueous cinnamon extract facilitates its proapoptotic activity is by increasing intracellular calcium, leading to loss of membrane potential and, eventually, apoptosis. In an independent study, authors observed an increase in intracellular calcium and depolarization of mitochondrial membrane potential, when SiHa cells were treated with 80 *μ*g/mL of aqueous cinnamon extract [68].

Histone deacetylase family member 8 (HDAC8) is implicated in many cancers, and an HDAC8 knockdown system showed decreased cancer progression of human colon, lung, and cervical cells. In an *in vitro* and *in silico* study, aqueous extract of cinnamon, cinnamic acid, cinnamyl alcohol, and cinnamaldehyde, were all shown to bind to the HDAC8 enzyme. The whole extract was much more efficient in inhibiting the enzyme compared to purified components [76]. Transformed cancerous cells are associated with increased activity of 26S proteasome, the protein degrading machinery. This increased activity facilitates cancer cell proliferation and survival. The aqueous cinnamon extract and one of its active components, procyanidin B2, were reported to have antiprotease activity (26S proteasome). These components inhibited the complete catalytic activity (all 3 activities of the 20S) and were selective for transformed cells only [62].

### 3.4. Anti-Inflammatory Activity

An infection-mediated immune response can be either innate when encountering the antigen for the first time or acquired when the response is generated from immunological memory [83]. The immune response is identified as “good” inflammation, which leads to an antigen-specific T-cell response; however, prolonged and uncontrolled inflammation can eventually lead to chronic inflammatory diseases and cancer [5]. The activated T-cell response is characterized by the production of cytokines such as interferon (IFN-*γ*), interleukin 2 (IL2), and interleukin 4 (IL4) and changes in the expression of signaling molecules [69]. In an immune response generated by activated T-cells, the aqueous cinnamon extract reduced the IFN-*γ* levels and inhibited p38, c-Jun amino-terminal kinases (JNK), extracellular signal-regulated kinases (ERK), signal transducer and activator of transcription 4 (STAT4), and signal transducer and activator of transcription 6 (STAT6), without affecting the inhibitor of kappa B (IkB*α*) [69]. Similarly, aqueous cinnamon extract downregulated lipopolysaccharide (LPS)-induced TNF-*α* levels in serum by inhibiting the activation of p38, JNK/ERK1 and 2, and IkB*α* [70]. LPS binds to receptors such as toll-like receptors which recognize a unique microbial pattern and elicit an immune response. The response is mediated by NFkB and MAP kinase signal pathways. In this *in vivo* and *in vitro* study [70], cinnamon aqueous extract at low doses was able to decrease TNF-*α* and IL-6 levels in the serum. Chronic inflammatory conditions are characterized by an IFN-*γ* driven initial response followed by the release of other inflammatory mediators. Aqueous extract of cinnamon as well as its polyphenol fraction exerts anti-inflammatory activities which result in decrease of IFN-*γ*, NFkB, and MAP kinase signal pathways and expression of IL6, IL8, and TNF-*α* [37, 70]. Another study using a cellular intestinal inflammation model revealed that aqueous cinnamon extract containing the active components, cinnamic acid and cinnamaldehyde, played a dual role in enhancing tight junction permeability and decreasing inflammatory modulators interleukin 6 (IL6), interleukin 8 (IL8), TNF-*α*, and NFkB. Cinnamon aqueous treated mice had better gut microbiota diversity than untreated counterpart [79].

### 3.5. Antioxidant Activity

One of the essential roles of aqueous cinnamon extract is its antioxidant activity. The extraction method affects the total phenol content and antioxidant potential.  Table 3 summarizes the effect of the extraction method and solvent on overall antioxidant capacity. To summarize, hydroethanolic solvent seems to perform better in terms of total phenol content and antioxidant activity than aqueous extract [42]. However, for the *in vivo* studies listed in the table, the preferred extraction solvent was water [49, 52]. For cinnamon aqueous extract to be useful as CAM, we have discussed its anticancer and anti-inflammatory role; in this section, we discuss the antioxidant role of aqueous extract which solidifies it as a CAM candidate. Effective chemotherapeutic agents, such as cis-diamminedichloroplatinum (CDDP), are associated with several side effects, one of which is increase in reactive oxygen species (ROS). Heme oxygenase-1 (HO-1) is upregulated in the kidneys to counteract the oxidative stress. In an *in vitro* study, Vero cells were treated with CDDP to induce toxicity and treatment with aqueous cinnamon extract which decreased CDDP-mediated ROS and helped in counteracting oxidative stress and decreased apoptotic cell death. One of the ways by which the extract brings about this effect is by upregulation of heme oxygenase-1 (HO-1) transcript levels without interfering with CDDP activity [74]. In another example, acetaminophen, an antipyretic and analgesic drug, if abused beyond therapeutic doses is associated with acute renal toxicity which results due to increased oxidative stress from increased ROS. In an *in vivo* study, the aqueous cinnamon extract has been shown to protect against acetaminophen-mediated cellular damage and apoptosis in renal cells by limiting/decreasing lipid peroxidation and apoptosis [87]. Small-molecule therapeutic agents are associated with acute toxicity when they exceed the recommended dose [74, 87], and the chances of toxicity are further increased when multiple therapeutic agents are administered together [61]. Diclofenac sodium (DFS), a nonsteroidal anti-inflammatory drug, is often consumed along with oxytetracycline (OTC) (an antibiotic), and the combination is associated with severe toxicities when abused. The toxicities are associated with increased ROS, decreased activity of antioxidant superoxide dismutase (SOD), and reduced glutathione (GSH). The antioxidant and anti-inflammatory properties of the aqueous cinnamon extract protected against the toxicity induced by DFS and OTC, individually or in combination, by increasing the proapoptotic factors, hepatic and renal caspase-3, and cyclooxygenase-II [61].

## 4. Active Components of Aqueous Cinnamon Extracts

### 4.1. Cinnamaldehyde

**Figure 5:**
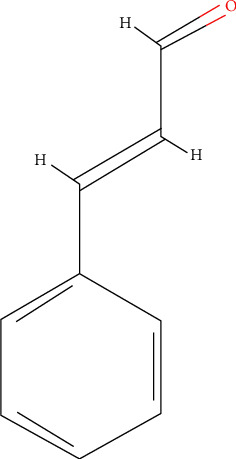
Structure of trans cinnamaldehyde [88].

Cinnamaldehyde (cinnamic aldehyde), a phenylpropanoid (Figure 5), occurs naturally as a trans-cinnamaldehyde and imparts the odor and flavor of cinnamon. It is one of the main components of cinnamon essential oils and is also present in the aqueous extract of cinnamon [61, 63]. Several studies have reported the anticancer and anti-inflammatory activities of cinnamaldehyde [65, 89]. The aqueous cinnamon extract containing cinnamaldehyde has been shown to exert higher cytotoxic activity than purified cinnamaldehyde against cancerous cell lines, suggesting that cinnamaldehyde, in conjunction with other polyphenols present in the aqueous cinnamon extract, plays a role in cancer cell cytotoxicity [66]. The aqueous cinnamon extract with cinnamaldehydes and polyphenols as the major bioactive compounds was cytotoxic to cancer cells. It downregulated human epidermal growth factor receptor-2 (EGFR-2) and matrix metalloproteinase (MMP-2) expression, affecting the invasion and metastasis of cervical cancer [68]. Liao et al. [90] showed that aqueous cinnamon extract and purified cinnamaldehyde exert their effect on TNF-*α*-induced signaling in endothelial cells. This effect is seen when cells are pretreated with either aqueous cinnamon extract or purified cinnamaldehyde. The mechanism of action is through blocking the degradation of the nuclear factor of kappa light polypeptide gene enhancer in B-cells inhibitor, alpha (I*κ*B*α*), when pretreatment is short term (up to 3 hrs). This results in a decrease in expression of intercellular adhesion molecule 1 (ICAM), vascular cell adhesion molecule 1 (VCAM), and NF-*κ*B signaling. Over long-term (up to 12 hrs.) pretreatment cinnamaldehyde causes induction of nuclear factor erythroid 2-related factor 2 (Nrf2)-related genes (including HO-1) which are cytoprotective. Cabello et al. [91], in their studies on A375 melanoma cells, demonstrated that cinnamaldehyde inhibited NFkB constitutive and TNF-*α*-induced transcriptional activity and suggested that Michael acceptor reactivity with this dietary electrophile is responsible for the observed effect.

Angiogenesis is central to the development and growth of tumor cells. Several small-molecule inhibitors and targeted therapies are aimed at inhibiting angiogenesis. The aqueous cinnamon extract decreased VEGF, a key molecule involved in angiogenesis, and was mediated by suppression of the HIF-1*α* gene via STAT3 and AKT. The decrease in VEGF and the resulting antiangiogenic effects were attributed to cinnamaldehyde, a major component of the extract [63]. Liu et al. [89] identified possible cinnamaldehyde targets in breast cancer using *in silico* and in an *in vitro* study demonstrated that cinnamaldehyde induced apoptosis, decreased proliferation, and reduced the ability of cells to migrate and invade.

### 4.2. Cinnamic Acid

**Figure 6:**
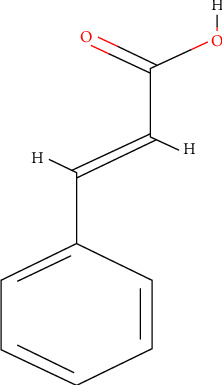
Structure of cinnamic acid [92].

Cinnamic acid is a naturally occurring unsaturated carboxylic acid with a widely present trans isomer. Similar to other components of cinnamon, cinnamic acid (Figure 6) is cytotoxic to cancerous cells. In an *in vitro* model, cinnamic acid inhibited cell growth and multiplication. It modulated the expression of *MMP2* and reduced the invasive capacity of cancer cells, suggesting its potential to reverse malignant human tumor cells to benign cells [93]. Cancerous cells upregulate antiapoptotic signaling cascades, leading to increased survival [4], which can be reversed by cinnamic acid. Niero et al. [94] showed that cinnamic acid induced apoptosis in HT-144 human melanoma cells by upregulating the proapoptotic machinery, caspase-3 activity, and Bax and downregulating antiapoptotic Bcl-2 protein.

Over the years, it has become apparent that overexpression of MMP proteins and malignant cancers are correlated. Yen et al. [95] demonstrated that cis and trans stereoisomers of cinnamic acid could inhibit the MMP-2 and 9 activity, decreasing the invasive ability of A549 cells. Moreover, MMP downregulation can be linked to transcription factors, such as NFkB, AP-1, and the mitogen-activated protein kinases (MAPK) pathway [96]. Kwon et al. [56] elucidated the anticancer activity of the cinnamon extract containing cinnamic acid and cinnamaldehyde as the major bioactive compounds in several transformed cell lines. Furthermore, in an intestinal epithelial cell and monocyte-macrophage coculture model, a cinnamon water extract downregulated the inflammatory markers, such as TNF-*α*, inducible nitric oxide synthase (iNOS), and COX-2, providing evidence for its anti-inflammatory activity. Even though the specific compound responsible for the anti-inflammatory effect was not identified in the study, cinnamic acid was one of the major bioactive compounds present in the extract.

### 4.3. Polyphenols

**Figure 7:**
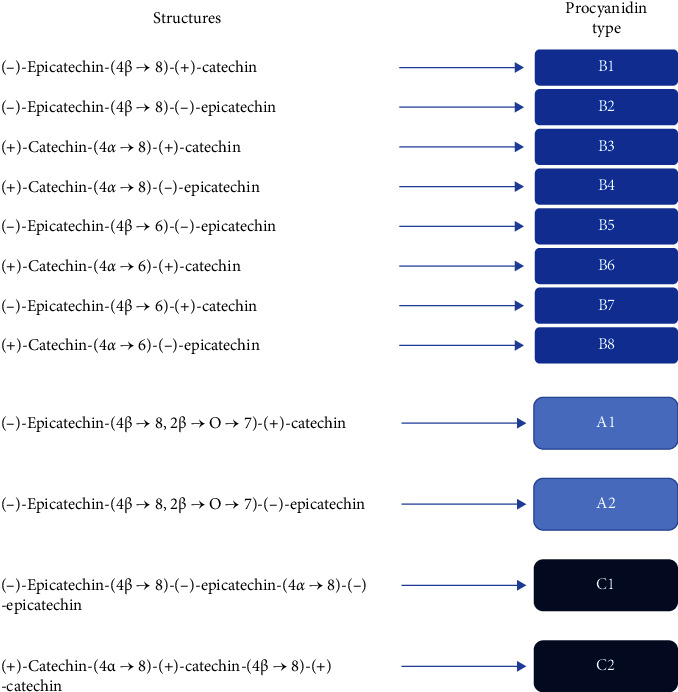
Procyanidin dimers and structures.

Polyphenols are naturally occurring organic compounds abundant in fruits, vegetables, tea, and spices. These secondary metabolites play a role in defense against pathogens and are known to protect against ultraviolet radiation [97]. Procyanidins comprising (epi) catechin monomeric units (Figure 7) play a protective role against cancer and inflammation [98, 99]. It has been reported that procyanidin tetramers and pentamers in *Cinnamomi cortex* (dried bark of *C. verum*) extract suppressed Nrf2-regulated enzyme activity in human lung cancer cell line A549 [100]. Nrf2 is a crucial cytoprotective transcription factor, and its overexpression induces resistance to chemotherapy, the most pressing dilemma in cancer therapy [101].

Angiogenesis is critical for cancer cells to flourish and propagate [102]. Of several molecules known to regulate angiogenesis, VEGF is the most important; it binds to its cognate receptors, VEGFR1 and VEGFR2, and initiates a signaling cascade leading to angiogenesis [102]. Therefore, VEGF and its receptors are attractive targets for cancer therapy [103]. The aqueous cinnamon extract could inhibit receptor kinase activity and downstream signaling of VEGFR2, suggesting the role of procyanidin trimers and tetramers in inhibiting VEGFR2 activity [67].

Proteosomes are another exciting target that has been identified for cancer therapy. Proteosome levels are elevated in tumor cells, which play an antiapoptotic role [62]. The procyanidin B2 component of the aqueous cinnamon extract was reported to selectively inhibit proteasome activity in cancer cells, decrease cell proliferation, and regulate genetic markers related to apoptosis and angiogenesis [2, 62].

Cancer and inflammation are intricately linked; therefore, targeting inflammatory markers can help in chronic pathologic inflammation and in cancers where inflammatory molecules exacerbate cancer progression. NFkB modulates the expression of several proinflammatory molecules and triggers inflammatory conditions. Targeting NFkB using Cinnulin PF®, an aqueous cinnamon extract containing cinnamic acid, phenolic acids, flavonoids, and procyanidins, primarily as trimers and tetramers, decreased the production of inflammatory cytokines in an lipopolysaccharide (LPS)-stimulated macrophage cell system, concomitantly with a significant reduction in NFkB activity (60.1%) [37].

## 5. Conclusion

The aqueous extract of cinnamon contains bioactive components that have significant protective effects against cancer and inflammatory conditions. The synergistic effects of these components have better therapeutic efficiency than when a purified component is used. Further studies are required to understand whether cinnamon extract should be used only as complementary and alternative medicine or if it could play a more significant role. A few studies have shown that cinnamon extract can work with chemotherapeutic agents; however, such studies are scarce. Further studies are essential to understand the potential of this multipurpose spice and identify its beneficial use as an adjunct therapy for various pathological conditions, especially cancer and chronic inflammation. Better characterization of the aqueous extract would help formulate an effective dose and regime for treatment. Collaborations between academics and the pharmaceutical industry could expedite this process and greatly help patients.

## Figures and Tables

**Table 1:** Advantages and limitations of various green extraction methods.

Extraction method	Extractants	Advantages	Limitations	Emerging scenarios	References
Hot-pressurized Liquid extraction (HPLE)	Water and aqueous alcohols (HPLE) and water (PHWE)	(i) Recovery of polyphenols is higher	(i) Low selectivity	Coupling with UAE and SFE	Panja [49] Chemat et al., [50] Kumar et al., [51] Gil-Martín et al. [52]
Pressurized hot water extraction (PHWE)		(ii) Low solvent requirement	(ii) Instrumentation cost		
Supercritical fluid extraction (SFE)	Supercritical CO_2_	(i) Suitable for extraction of nonpolar or mid to low polar components	(i) Whole extracts cannot be achieved due to selectivity	Coupling with UAE	
Microwave-assisted extraction (MAE)	Water, ethanol, and methanol	(i) Most suitable for extraction of flavonoids and phenolic acids	(i) Limitation of the technology as it is can be used for specific compounds	Vacuum microwave aqueous assisted extraction or VMAAE	
		(ii) Low solvent requirement			
Ultrasound assisted extraction (UAE)	Water and aqueous alcohols	(i) Cost effectiveness	(i) High power UAE can cause degradation of desired species	Coupling with MAE and SFE	
		(ii) Good for thermolabile/unstable components			
Enzyme assisted extraction (EAE)	Water	(i) Low energy requirement	(i) Time consuming	Coupling with high hydrostatic pressure (HHP)	
		(ii)Reduced/no degradation of desired species as the temperature working range is low			
Pulsed electric field extraction (PEF)	Not applicable	(i) Suitable for useful for thermolabile components	(i) Not yet identified	PEF with solvent extraction	

**Table 2:** Methods for preparing an aqueous extract of cinnamon, its effects, and significant bioactive compounds present in the extract.

Aqueous extraction methods	Concentrations used	Cinnamon species and the plant part used	Results	Major bioactive compounds identified/tested	References
Ground cinnamon extracted in hot water extractor for 3 h, supernatant freeze-dried after sterile filtration	Concentration (*in vivo* study)-10 mg/dose (400 *μ*g/g of body weight)	*C. cassia* (bark)	Study demonstrates the role of aqueous cinnamon extract as an anticancer agent	Not stated	Kwon et al. [65]
	Concentrations (*in vitro* study)-0.3 and 0.5 mg/mL		Treatment with the aqueous cinnamon extract resulted in decreased neovascularization and increased cytolytic activity of CD8+ T cells in a mouse melanoma model		

Ground cinnamon (5 g) was added to 50 mL nanopure water at 40°C (round bottom flask in a mineral oil bath at 40°C), stirred rapidly for 10 min. Centrifuged (10 min at 12,000 ×g, 4°C), placed in a prechilled beaker in an ice bath, and again stirred for 30 min. Filtered, flash-frozen, and then lyophilized. Yield was approx. 7% of the starting material	Concentrations (*in vitro* studies)-up to 0.22 mg/mL of cinnamon extract, up to 100 *μ*M of a-linked proanthocyanidin trimer and cinnamaldehyde	*C. zeylanicum*	Study demonstrates the role of the aqueous extract of cinnamon in inhibition of the aggregation of human tau *in vitro* (a critical process in Alzheimer's disease)	A-linked proanthocyanidin trimer and cinnamaldehyde	Peterson et al. [55]

50 mg/1 mL aqueous solution of cinnamon powder, heated at 15 PSI and 121°C for 20 min. The resulting extract was centrifuged (10,000 rpm for 10 min), and the supernatant was filter sterilized and stored at −80°C	Concentrations (*in vitro* studies)-up to 1.28 mg/mL of cinnamon extract and up to 320 *μ*M of cinnamaldehyde	*C. zeylanicum* (bark)	Study demonstrates the role of aqueous cinnamon extract as an anticancer agent	Polyphenols and cinnamaldehyde	Singh et al. [66]

Ground cinnamon extracted in hot water extractor for 3 h, supernatant freeze-dried after sterile filtration	Cinnamon water extract concentration (*in vivo* study)-10 mg/dose (400 *μ*g/g of mouse weight)	*C. cassia* (bark)	Study demonstrates the role of aqueous cinnamon extract as an anticancer agent focusing on the induction of apoptosis	Not stated	Kwon et al. [56]
	Concentrations (*in vitro* study)-0.3 and 0.5 mg/mL		Observed effects were a reduction in NF*κ*B and AP1 expression and downstream antiapoptotic genes		

Ground cinnamon powder resuspended in sterile water and heated at 70°C for 1 h, centrifuged, and used after filtration	Cinnamon water extract concentrations (*in vitro* study)-up to 75 *μ*g/ml	*C. zeylanicum*	Study demonstrates the role of aqueous cinnamon extract as an anticancer agent via inhibition of angiogenesis	Procyanidin oligomers	Lu et al. [67]
			Focus on VEGF inhibitors present in the aqueous cinnamon extract		

Ground cinnamon was extracted (1 : 16 ratio of cinnamon to water) in a hot water extractor, centrifuged at 13000 rpm for 15 min, and used after sterile filtration	Cinnamon water extract concentrations (*in vitro* study)-up to 80 *μ*g/ml	*C. cassia* (bark)	Study demonstrates the role of the aqueous cinnamon extract as an anticancer agent	Not stated	Koppikar et al. [68]
			Observed effects were decreased growth rate of SiHa cells, decreased expression of MMP-2, and downregulation of Her-2 expression in SiHa cells		

A 400 g bark was soaked in an equal volume of water (overnight), sonicated for 1 h, filtered, and freeze-dried at −70°C. The dried material was reconstituted in phosphate-buffered saline and sterile filtered before use. Yield was 0.7% of the starting material weight	Cinnamon water extract concentrations (ex vivo study)-up to 200 *μ*g/ml	*C. cassia* (bark)	Study demonstrates the role of the aqueous extract of cinnamon in inflammatory disorders	Not stated	Lee et al. [69]
	Cinnamon water extract concentrations (*in vivo* study)-up to 1 g/kg of body weight		Observed effects were downregulation of IFN-*γ* expression and inhibition of downstream signaling pathways such as p38, JNK, and STAT4		

Ground cinnamon soaked in one volume of water (48 h at room temperature), sonicated (1 h), filtered, and freeze-dried at −70°C. Yield was approximately 3.62%	Cinnamon water extract concentrations (*in vitro* study)-up to 100 *μ*g/ml	*C. cassia* (bark)	Study demonstrates the role of the aqueous extract of cinnamon as an anti-inflammatory agent	Polyphenols	Hong et al. [70]
	Concentrations (*in vivo* study)-up to 500 mg/kg of body weight		In an *in vivo* study using a mouse model, treatment with an aqueous extract of cinnamon decreased TNF-*α* and IL-6 release in serum when stimulated with LPS. I*κ*B*α* degradation and MAPK activation were observed in LPS-stimulated macrophages *in vitro*		

A total of 5 g ground cinnamon was soaked in 50 mL water, incubated for 5 h at 40°C, the extract centrifuged (7000 ×g for 10 min), and the supernatant stored at −20°C after lyophilization	Cinnamon water extract and proanthocyanidins concentrations (*in vitro* study)-up to 82 *μ*g/ml	*C. zeylanicum* (bark)	Role of aqueous extract of cinnamon in managing type 2 diabetes mellitus	Proanthocyanidins, cinnamic acid, coumarin, and cinnamaldehyde	Jiao et al. [71]
			The aqueous extract inhibited the aggregation of human islet amyloid polypeptide (hIAPP), which is one of the causes of type 2 diabetes mellitus		

Ground cinnamon was added to distilled water (10 mL/g), incubated, and stirred for 4 h, followed by boiling for 2 h, centrifuging (7500 ×g for 30 min), filtering, and lyophilizing	Cinnamon water extract concentrations (*in vitro* study)-up to 8 mg/ml	Not available	This study demonstrated the anticancer activity of the aqueous extract of cinnamon on transformed cell lines (HeLa, MCF-7, and MDA-MB234)	Not available	Ariaee-Nasab et al. [72]

A total of 100 g of ground cinnamon was soaked in one liter of water (48 h at room temperature), sonicated (1 hour), filtered, and lyophilized	Cinnamon water extract concentrations (*in vitro* study)-up to 100 *μ*g/ml	*C. cassia* (bark)	This study demonstrated the role of the aqueous extract of cinnamon in preventing the development of atherosclerotic lesions and associated inflammation	Cinnamon, cinnamyl alcohol, cinnamic acid, cinnamaldehyde, coniferyl aldehyde, coumarin polyphenols, catechin, and epicatechin	Kang et al. [73]
			The aqueous extract interfered with monocyte differentiation (decreasing the expression of CD11b, CD36) and macrophage scavenger activity		

A total of 100 mg of ground cinnamon was dissolved in 1000 mL water, boiled (100°C for 30 min), centrifuged, filtered, and lyophilized. Dissolved in DMSO after lyophilization	Cinnamon water extract concentrations (*in vitro* study)-up to 50 *μ*g/ml	*C. cassia* (bark)	This study demonstrated the role of the aqueous extract of cinnamon against chemotherapeutic-induced toxicity	Phenylpropanoids, phenols, *β*-cadinene, *α*-murolene, *α*-cadinene, and hydrocarbons	ElKady and Ramadan [74]
			The aqueous extract prevented the activation of various cellular mechanisms mediating apoptosis without compromising the anticancer efficiency of cis-diamine-dichloro platinum (CDDP)		

A total of 80 g of dried cinnamon powder was added to 400 mL water (stirred on a magnetic stirrer at 80°C for 15 h), filtered, and stored in a −80°C freezer until completely frozen. Freeze-dried for one week, weighed, and stored at room temperature. Yield was 2.54%	Cinnamon water extract concentrations (*in vitro* study)-up to 6.25 mg/ml	*C. zeylanicum* (bark)	This study demonstrated the role of the aqueous extract of cinnamon as an anticancer agent	Cinnamaldehyde, cinnamyl alcohol, eugenol, methoxy cinnamaldehyde, hexadecenal, and hexadecenoic acid	Abd Wahab and Adzmi [41]
			Cytotoxic effect of the aqueous extract towards MCF-7 cell line		

Cinnamon powder (500 g) was dissolved in distilled water (500 ml, boiled for 3 h, filtered, and freeze-dried (in a vacuum evaporator). Sterilized by radiation	Cinnamon water extract concentrations (*in vivo* study)-500 mg of cinnamon/kg body mass/day	*C. cassia* (bark)	This study demonstrated the role of the aqueous extract of cinnamon as an anticancer agent	Not available	Ezzat et al. [75]
			The study focused on the effect of the aqueous extract of cinnamon in preventing oral carcinogenesis induced by 7,12-di-methylbenz[a]anthracene (DMBA) hamster cheek pouch (HCP) mucosa		

Powdered cinnamon was extracted in water (1 : 16 w/v) in a hot water extractor. The extract was centrifuged (13000 rpm for 15 min), and the filter sterilized	Cinnamon water extract concentrations (*in vitro* study)-up to 80 *μ*g/ml	*C. cassia* (bark)	This study demonstrated the inhibitory potential of the aqueous extract of cinnamon against histone deacetylase family member 8 (HDAC8), which is linked with various cancers	Cinnamaldehyde, cinnamic acid, cinnamyl alcohol	Raina et al. [76]
			The potency of aqueous extract was much better than individual bioactive components, indicating that these components might be working synergistically to induce inhibition		

Cinnamon powder was dissolved in water (70°C, 1 h), centrifuged (13,000 rpm, 10 min), filtered, and lyophilized under vacuum	Cinnamon water extract concentrations (*in vitro* study) - up to 32 *μ*g/ml	*C. zeylanicum*	Role of aqueous extract of cinnamon as anticancer and antiangiogenic agent	Cinnamaldehyde and procyanidins	Zhang et al. [77]
	Cinnamon water extract concentrations (*in vivo* study in mice) -0.3 mg of cinnamon extract/g body weight		Cinnamon aqueous extract suppressed HIF-1*α* gene expression and protein synthesis resulting in inhibition of VEGF production in the cancer cells. Treatment also inhibited the expression and phosphorylation of downstream signal transduction molecules such as STAT3 and AKT		

Five grams of ground cinnamon were added to 25 mL of water and kept in a water bath (55°C for 3 h), vortexed (every 30 min), followed by centrifugation (6300 ×g for 15 min). Supernatant filtered and stored	Cinnamon water extract concentrations (*in vitro* study)-up to 25 mg/ml	Not available	Role of aqueous cinnamon extract and one of its bioactive components, procyanidinB2 (PCB2), in inhibiting the proteasome activity and suppression of prostate cancer cell growth	procyanidinB2 (PCB2)	Gopalakrishnan et al. [62]
			The aqueous treatment resulted in a decrease in markers involved in the survival of cells		

A total of 10 g of the dried powdered soaked in 100 mL water and boiled (2 h), filtered. Filtrate dried by heating overnight at 80°C in an oven	Cinnamon water extract concentrations (*in vivo* study in rats) -400 mg/kg orally once a day for 15 days	*C. zeylanicum* (bark)	Role of aqueous extract of cinnamon in as an antioxidant	Cinnamaldehyde, cinnamic acid, and eugenol	Abdeen et al. [78]
			Results showed that when rats were pretreated with the aqueous cinnamon extract, it led to decreased acetaminophen-induced cellular alterations and apoptosis in healthy cells		

Ground cinnamon (30g) was added to water (1 : 10, w/v) and extracted using a subcritical water extraction device at 110°C for 40 minutes, with a pressure of 50 bar. The extract was centrifuged at 4°C (11 000 ×g for 5 min), filtered, and lyophilized	Cinnamon subcritical water extract concentrations (*in vitro* study)-up to 100 *μ*g/ml	*C. japonicum* (bark)	Role of aqueous extract of cinnamon in cellular intestinal inflammation model	Cinnamic acid and cinnamaldehyde	Kim and Kim [79]
			Significant downregulation of the PGE2, TNF-*α*, cytokines, and NFkB activity was reported		

Ground cinnamon soaked in water (1 : 20, w/v for 48 h), filtered, placed in rotary devices (3-4 h), condensed extracts obtained were centrifuged (10,000 rpm, 15 min), the supernatant was sterile filtered and stored at −80°C	Cinnamon water extract concentrations (*in vitro* study)-up to 1.3 mg/ml	Not available	Role of the aqueous cinnamon extract as an anticancer agent in treating oral squamous cell carcinoma	Not available	Dehghani Nazhvani et al. [9]

An aqueous solution of 10 mg/mL of ground cinnamon was prepared and kept at 4°C in the dark after filtering	Cinnamon water extract concentrations (*in vitro* study)-up to 500 *μ*g/ml	*C. burmannii* (bark) commercialized as Cinnulin® PF	Role of aqueous cinnamon extract as an antiinflammatory agent to treat periodontal diseases	Phenolic acids, flavonoids (flavanols, anthocyanins, flavan-3-ols), and procyanidins, cinnamon fraction, cinnamic acid was the most abundant phenolic acid	Ben Lagha et al. [37]
			The polyphenolic fraction of aqueous cinnamon extract reduced IL-6, IL-8, and TNF-*α* secretion		

The aqueous solution was prepared at 10%, boiled for 2 h at 100°C, and dried overnight (80°C) after filtering. Yield was 20% w/w	Cinnamon water extract concentrations (*in vivo* study in rats)-300 mg/kg daily for 15 days	*C. zeylanicum* (bark)	Role of the aqueous extract of cinnamon anti-inflammatory and antioxidant agent	Phenols, flavonoids, and tannins	Elshopakey and elazab 2021 [61]
			Aqueous extract of cinnamon protected against diclofenac sodium (DFS) and oxytetracycline (OTC) toxicity in a rat model		

**Table 3:** Antioxidant activity of cinnamon extracts.

Extraction method	Species	Total phenol	Antioxidant activity	Study outcome	References
2.50 g cinnamon in 60 ml distilled water and placed in a reflux system for extraction. The extract was cooled, filtered and concentrated to 50 mL. Extract final concentration 0.05 g/mL	*C. zeylanicum* (bark)	Total polyphenol content (mg GAE/g d w) was 11.60 ± 1.9	IC50 value (*μ*g/mL) was 95.08 ± 0.06 (DPPH assay)	Doxorubicin treatment leads to increase reactive oxygen species (ROS). *In vivo* study in Wistar rats showed that pretreatment of cinnamon extract led to restoration of antioxidants enzymes and aqueous extract also had radical scavenging ability	Sandamali et al. [84]

500 g cinnamon fruits powder (defatted) dried, powdered, extracted for 4 hours in various solvents including water. The yield for water extract was 19.35 g	*C. zeylanicum* (fruit)	Not available	At 12.5 ppm, protocatechuic acid, cinnamtannin B-1, urolignoside, rutin, quercetin-3-O-R-L-rhamnopyranoside had 77.3, 39.3, 30.4, 44.7, 60.3% free radical scavenging activities, respectively	High phenolic content and antioxidant activity in the water extract. protocatechuic acid, cinnamtannin B-1, urolignoside, rutin, and quercetin-3-O-*α*-L-rhamnopyranoside were purified from the extract	Jayaprakasha et al. [85]

Defatted cinnamon powder was extracted in water by boiling, followed by filtration and vacuum dried	*C. verum* (bark)	Not available	Effect of water and ethanol extract was compared by measuring the antioxidant activity of several liver enzymes in treated vs. untreated animal groups. (lipid peroxide product (malendialdlyde), catalase, superoxide dismutase (SOD), and serum aminotransferase enzymes (ALT and AST))	Ethanol extract has better radical scavenging activity then water extract	Moselhy and Ali [86]

Dried cinnamon powder was extracted in various solvents (water, ethanol, methanol, acetone and ethyl acetate). The extraction was optimized for solute to solvent ratio, extraction time and temperature and finally the phenolic content and radical scavenging activity	*C. cassia*	1.918 ± 0.528 mmol/g (under optimized condition)	14.337 ± 4.662 mmol of TEAC. (under optimized condition)	Optimized conditions-60% (v/v) ethanol, 1 : 20 sample weight ratio for 90 mins at 50°C	Dvorackova et al. [42]

CD8: cluster of differentiation 8, NF-kB: nuclear factor kappa light chain enhancer of activated B cells, AP1: activator protein 1, VEGF: vascular endothelial growth factor, MMP-2: matrix metalloproteinase-2 (type IV collagenase, gelatinase A), HER2: human epidermal growth factor receptor 2, IFN-*γ*: interferon gamma, p38: p38 mitogen-activated protein kinases, JNK: -c-jun N-terminal kinase, STAT4: signal transducer and activator of transcription 4, TNF-*α*: tumor necrosis factor alpha, IL6: interleukin-6, LPS: lipopolysaccharides, IkB*α* (nuclear factor of kappa light polypeptide gene enhancer in B-cell inhibitor, alpha), MAPK: mitogen-activated protein kinases, CD11b: cluster of differentiation 11b, CD36: cluster of differentiation 36, STAT3: signal transducer and activator of transcription 3, AKT: it refers to protein kinase B (PKB), PGE2: prostaglandin E2, and IL-8: interleukin-8.

## Data Availability

No data were generated to support the findings of this study.

## References

[1] Doocey C. M., Finn K., Murphy C., Guinane C. M. (2022). The impact of the human microbiome in tumorigenesis, cancer progression, and biotherapeutic development. *BMC Microbiology*.

[2] Cooper G. M. (2000). The development and causes of cancer. *The Cell: A Molecular Approach*.

[3] B Vendramini-Costa D., E Carvalho J. (2012). Molecular link mechanisms between inflammation and cancer. *Current Pharmaceutical Design*.

[4] Hanahan D. (2022). Hallmarks of cancer: new dimensions. *Cancer Discovery*.

[5] Rossi J. F., Lu Z. Y., Massart C., Levon K. (2021). Dynamic immune/inflammation precision medicine: the good and the bad inflammation in infection and cancer. *Frontiers in Immunology*.

[6] Greten F. R., Grivennikov S. I. (2019). Inflammation and cancer: triggers, mechanisms, and consequences. *Immunity*.

[7] Zhao H., Wu L., Yan G. (2021). Inflammation and tumor progression: signaling pathways and targeted intervention. *Signal Transduction and Targeted Therapy*.

[8] Ho W. J., Jaffee E. M., Zheng L. (2020). The tumour microenvironment in pancreatic cancer—clinical challenges and opportunities. *Nature Reviews Clinical Oncology*.

[9] Dehghani Nazhvani A., Sarafraz N., Askari F., Heidari F., Razmkhah M. (2020). Anti-cancer effects of traditional medicinal herbs on oral squamous cell carcinoma. *Asian Pacific Journal of Cancer Prevention*.

[10] Munn L. L. (2017). Cancer and inflammation. *Wiley Interdiscip Rev Syst Biol Med*.

[11] Nakamura H., Takada K. (2021). Reactive oxygen species in cancer: current findings and future directions. *Cancer Science*.

[12] Łukaszewicz-Zając M., Mroczko B. (2021). Circulating biomarkers of colorectal cancer (CRC)—their utility in diagnosis and prognosis. *Journal of Clinical Medicine*.

[13] Zhou W., Yang L., Nie L., Lin H. (2021). Unraveling the molecular mechanisms between inflammation and tumor angiogenesis. *Am J Cancer Res*.

[14] Crusz S. M., Balkwill F. R. (2015). Inflammation and cancer: advances and new agents. *Nature Reviews Clinical Oncology*.

[15] Lan T., Chen L., Wei X. (2021). Inflammatory cytokines in cancer: comprehensive understanding and clinical progress in gene therapy. *Cells*.

[16] Marei H. E., Althani A., Afifi N. (2021). p53 signaling in cancer progression and therapy. *Cancer Cell International*.

[17] Kennedy M. C., Lowe S. W. (2022). Mutant p53: ’it’s not all one and the same. *Cell Death &amp;amp; Differentiation*.

[18] Bhatta B., Luz I., Krueger C. (2021). Cancer cells shuttle extracellular vesicles containing oncogenic mutant p53 proteins to the tumor microenvironment. *Cancers*.

[19] Capaci V., Mantovani F., Del Sal G. (2020). Amplifying tumor–stroma communication: an emerging oncogenic function of mutant p53. *Frontiers Oncology*.

[20] Jing X., Yang F., Shao C. (2019). Role of hypoxia in cancer therapy by regulating the tumor microenvironment. *Molecular Cancer*.

[21] Shi D., Jiang P. (2021). A different facet of p53 function: regulation of immunity and inflammation during tumor development. *Frontiers in Cell and Developmental Biology*.

[22] Almatroodi S. A., Alsahli M. A., Almatroudi A. (2021). Potential therapeutic targets of quercetin, a plant flavonol, and its role in the therapy of various types of cancer through the modulation of various cell signaling pathways. *Molecules*.

[23] Balneaves L. G., Watling C. Z., Hayward E. N. (2021). Addressing complementary and alternative medicine use among individuals with cancer: an integrative review and clinical practice guideline. *Journal of the National Cancer InstituteJournal of the National Cancer Institute*.

[24] Asiimwe J. B., Nagendrappa P. B., Atukunda E. C. (2021). Prevalence of the use of herbal medicines among patients with cancer: a systematic review and meta-analysis. *Evidence-based Complementary and Alternative Medicine*.

[25] George B. P., Chandran R., Abrahamse H. (2021). Role of phytochemicals in cancer chemoprevention: insights. *Antioxidants*.

[26] Kubczak M., Szustka A., Rogalińska M. (2021). Molecular targets of natural compounds with anti-cancer properties. *International Journal of Molecular Sciences*.

[27] Dini I., Laneri S. (2021). Spices, condiments, extra virgin olive oil and aromas as not only flavorings, but precious allies for our wellbeing. *Antioxidants*.

[28] Tagde P., Tagde P., Islam F. (2021). The multifaceted role of curcumin in advanced nanocurcumin form in the treatment and management of chronic disorders. *Molecules*.

[29] Kammath A. J., Nair B., Nath L. R. (2021). Curry versus cancer: potential of some selected culinary spices against cancer with *in vitro, in vivo*, and human trials evidences. *Journal of Food Biochemistry*.

[30] Singh N., Rao A. S., Nandal A. (2021). Phytochemical and pharmacological review of *Cinnamomum verum* J. Presl-a versatile spice used in food and nutrition. *Food Chemistry*.

[31] Moosavi-Movahedi A. A. (2021). *Rationality and Scientific Lifestyle for Health*.

[32] Ribeiro-Santos R., Andrade M., Madella D. (2017). Revisiting an ancient spice with medicinal purposes: cinnamon. *Trends in Food Science & Technology*.

[33] Kawatra P., Rajagopalan R. (2015). Cinnamon: mystic powers of a minute ingredient. *Pharmacognosy Research*.

[34] Gilani S., Najafpour G. (2022). Evaluation of the extraction process parameters on bioactive compounds of cinnamon bark: a comparative study. *Process Biochemistry (Barking, UK)*.

[35] Rao M. V., Sengar A. S., Sunil C. K., Rawson A. (2021). Ultrasonication-A green technology extraction technique for spices: a review. *Trends in Food Science & Technology*.

[36] Souza V. B., Holkem A. T., Thomazini M. (2021). Study of extraction kinetics and characterization of proanthocyanidin‐rich extract from Ceylon cinnamon (*Cinnamomum zeylanicum*). *Journal of Food Processing and Preservation*.

[37] Ben Lagha A., Azelmat J., Vaillancourt K., Grenier D. (2021). A polyphenolic cinnamon fraction exhibits anti-inflammatory properties in a monocyte/macrophage model. *PLoS One*.

[38] Larasati Y. A., Meiyanto E. (2018). Revealing the potency of cinnamon as an anti-cancer and chemopreventive agent. *Indonesian Journal of Cancer Chemoprevention*.

[39] Sadeghi S., Davoodvandi A., Pourhanifeh M. H. (2019). Anti-cancer effects of cinnamon: insights into its apoptosis effects. *European Journal of Medicinal Chemistry*.

[40] Muhammad D. R. A., Dewettinck K. (2017). Cinnamon and its derivatives as potential ingredient in functional food—a review. *International Journal of Food Properties*.

[41] Abd Wahab W., Adzmi A. N. (2017). The investigation of cytotoxic effect of Cinnamomum zeylanicum extracts on human breast cancer cell line (MCF-7). *Sci Herit J*.

[42] Dvorackova E., Snoblova M., Chromcova L., Hrdlicka P. (2015). Effects of extraction methods on the phenolic compounds contents and antioxidant capacities of cinnamon extracts. *Food Science and Biotechnology*.

[43] Rachid A. P., Moncada M., Mesquita M. F., Brito J., Bernardo M. A., Silva M. L. (2022). Effect of aqueous cinnamon extract on the postprandial glycemia levels in patients with type 2 diabetes mellitus: a randomized controlled trial. *Nutrients*.

[44] Madhushika K. T. S., Bulugahapitiya V. P. (2022). Variation of bioactive secondary metabolites in Ceylon cinnamon (Cinnamomum zeylanicum Blume) in different climatic conditions, maturity status and propagation types. *Journal of Agriculture and Value Addition*.

[45] Wijeweera A. A., Hewage J. W., Jayasinghe G. G., Wadumethrige S. H., Hettiarachchi S. R., Wijesinghe K. G. G. (2022). Maturity dependence of quality, quantity and chemical constituents of bark and leaf oil of Ceylon Cinnamon (Cinnamomum zeylanicum Blume). *Ruhuna Journal of Science*.

[46] Klejdus B., Kováčik J. (2016). Quantification of phenols in cinnamon: a special focus on “total phenols” and phenolic acids including DESI-Orbitrap MS detection. *Industrial Crops and Products*.

[47] Bernard D., Kwabena A., Osei O., Daniel G., Sandra A., Elom S. (2014). The effect of different drying methods on the phytochemicals and radical scavenging activity of Ceylon Cinnamon (Cinnamomum zeylanicum) plant parts. *European Journal of Medicinal Plants*.

[48] Chemat F., Vian M. A., Cravotto G. (2012). Green extraction of natural products: concept and principles. *International Journal of Molecular Sciences*.

[49] Panja P. (2018). Green extraction methods of food polyphenols from vegetable materials. *Current Opinion in Food Science*.

[50] Chemat A. V., Ravi K., Hilali P. (2019). Review of alternative solvents for green extraction of food and natural products: panorama, principles, applications and prospects. *Molecules*.

[51] Kumar M., Dahuja A., Tiwari S. (2021). Recent trends in extraction of plant bioactives using green technologies: a review. *Food Chemistry*.

[52] Gil-Martín E., Forbes-Hernández T., Romero A., Cianciosi D., Giampieri F., Battino M. (2022). Influence of the extraction method on the recovery of bioactive phenolic compounds from food industry by-products. *Food Chemistry*.

[53] Castro-Puyana M., Marina M. L., Plaza M. (2017). Water as green extraction solvent: principles and reasons for its use. *Current Opinion in Green and Sustainable Chemistry*.

[54] Jeyaratnam N., Nour A. H., Kanthasamy R., Nour A. H., Yuvaraj A. R., Akindoyo J. O. (2016). Essential oil from Cinnamomum cassia bark through hydrodistillation and advanced microwave assisted hydrodistillation. *Industrial Crops and Products*.

[55] Peterson D. W., George R. C., Scaramozzino F. (2009). Cinnamon extract inhibits tau aggregation associated with ’Alzheimer’s disease *in vitro*. *Journal of Alzheimer’s Disease*.

[56] Kwon H. K., Hwang J. S., So J. S. (2010). Cinnamon extract induces tumor cell death through inhibition of NF-*κ*B and AP1. *BMC Cancer*.

[57] Silva M. L., Bernardo M. A., Singh J., de Mesquita M. F. (2022). Cinnamon as a complementary therapeutic approach for dysglycemia and dyslipidemia control in type 2 diabetes mellitus and its molecular mechanism of action: a review. *Nutrients*.

[58] Yaseen O. K., Mohammed M. T. (2022). Cinnamon zeylanicum aqueous improves some oxidative stress biomarkers in diabetic type 2 induced by streptozotocin in adult male albino mice. *Egyptian Journal of Chemistry*.

[59] El-Ashmawy N. E., Khedr E. G., Alfeky N. H., Ibrahim A. O. (2022). Upregulation of GLUT4 and PI3K, and downregulation of GSK3 mediate the anti-hyperglycemic effects of proanthocyanidins. *Medicine International*.

[60] Senevirathne B. S., Jayasinghe M. A., Pavalakumar D., Siriwardhana C. G. (2022). Ceylon cinnamon: a versatile ingredient for futuristic diabetes management. *Journal of Future Foods*.

[61] Elshopakey G. E., Elazab S. T. (2021). Cinnamon aqueous extract attenuates diclofenac sodium and oxytetracycline mediated hepato-renal toxicity and modulates oxidative stress, cell apoptosis, and inflammation in male albino rats. *Veterinary Sciences*.

[62] Gopalakrishnan S., Ediga H. H., Reddy S. S., Reddy G. B., Ismail A. (2018). Procyanidin-B2 enriched fraction of cinnamon acts as a proteasome inhibitor and anti-proliferative agent in human prostate cancer cells. *IUBMB Life*.

[63] Zhang K., Han E. S., Dellinger T. H. (2017). Cinnamon extract reduces VEGF expression via suppressing HIF-1*α* gene expression and inhibits tumor growth in mice: cinnamon extract inhibits VEGF expression. *Molecular Carcinogenesis*.

[64] Muzolf-Panek M., Stuper-Szablewska K. (2021). Comprehensive study on the antioxidant capacity and phenolic profiles of black seed and other spices and herbs: effect of solvent and time of extraction. *Food Measure*.

[65] Kwon H. K., Jeon W. K., Hwang J. S. (2009). Cinnamon extract suppresses tumor progression by modulating angiogenesis and the effector function of CD8+ T cells. *Cancer Letters*.

[66] Singh R., Koppikar S. J., Paul P., Gilda S., Paradkar A. R., Kaul-Ghanekar R. (2009). Comparative analysis of cytotoxic effect of aqueous cinnamon extract from Cinnamomum zeylanicum bark with commercial cinnamaldehyde on various cell lines. *Pharmaceutical Biology*.

[67] Lu J., Zhang K., Nam S., Anderson R. A., Jove R., Wen W. (2010). Novel angiogenesis inhibitory activity in cinnamon extract blocks VEGFR2 kinase and downstream signaling. *Carcinogenesis*.

[68] Koppikar S. J., Choudhari A. S., Suryavanshi S. A., Kumari S., Chattopadhyay S., Kaul-Ghanekar R. (2010). Aqueous cinnamon extract (ACE-c) from the bark of Cinnamomum cassia causes apoptosis in human cervical cancer cell line (SiHa) through loss of mitochondrial membrane potential. *BMC Cancer*.

[69] Lee B. J., Kim Y. J., Cho D. H., Sohn N. W., Kang H. (2011). Immunomodulatory effect of water extract of cinnamon on anti-CD3-induced cytokine responses and p38, JNK, ERK1/2, and STAT4 activation. *Immunopharmacology and Immunotoxicology*.

[70] Hong J. W., Yang G. E., Kim Y. B., Eom S. H., Lew J. H., Kang H. (2012). Anti-inflammatory activity of cinnamon water extract *in vivo* and *in vitro*LPS-induced models. *BMC Complementary and Alternative Medicine*.

[71] Jiao L., Zhang X., Huang L. (2013). Proanthocyanidins are the major anti-diabetic components of cinnamon water extract. *Food and Chemical Toxicology*.

[72] Ariaee-Nasab N., Vahedi Z., Vahedi F. (2014). Inhibitory effects of cinnamon water extract on human tumor cell lines. *Asian Pacific Journal of Tropical Disease*.

[73] Kang (2014). Effect of cinnamon water extract on monocyte-to-macrophage differentiation and scavenger receptor activity. *BMC Complementary and Alternative Medicine*.

[74] ElKady A. I., Ramadan W. S. (2016). The aqueous extract of cinnamon bark ameliorated cisplatin-induced cytotoxicity in Vero cells without compromising the anticancer efficiency of cisplatin. *Biomedical Papers*.

[75] Ezzat, Samah K. (2017). Effects of aqueous cinnamon extract on chemically-induced carcinoma of hamster cheek pouch mucosa. *Biochemistry and Biophysics Reports*.

[76] Raina P., Patil M., Choudhari A., Pandita S., Islam M. A., Kaul-Ghanekar R. (2017). Cinnamaldehyde, cinnamic acid, and cinnamyl alcohol, the bioactives of Cinnamomum cassia exhibit HDAC8 inhibitory activity: an *in vitro* and *in silico* study. *Pharmacognosy Magazine*.

[77] Zhang (2017). Cinnamon Extract Reduces VEGF Expression via Suppressing HIF-1*α* Gene Expression and Inhibits Tumor Growth in Mice: CINNAMON EXTRACT INHIBITS VEGF EXPRESSION. *Molecular Carcinogenesis*.

[78] Abdeen (2017). Protective effect of cinnamon against acetaminophen-mediated cellular damage and apoptosis in renal tissue. *Environmental Science and Pollution Research*.

[79] Kim M. S., Kim J. Y. (2019). Cinnamon subcritical water extract attenuates intestinal inflammation and enhances intestinal tight junction in a Caco-2 and RAW264.7 Co-Culture Model. *Food & Function*.

[80] Ansari M. J., Bokov D., Markov A. (2022). Cancer combination therapies by angiogenesis inhibitors; a comprehensive review. *Cell Communication and Signaling*.

[81] Rajabi S., Maresca M., Yumashev A. V., Choopani R., Hajimehdipoor H. (2021). The most competent plant-derived natural products for targeting apoptosis in cancer therapy. *Biomolecules*.

[82] Varalakshmi B., Anand V., Karpagam T. (2017). Phytochemical analysis of cinnamomum zeylanicum bark and molecular docking of procyanidin B2 against the transcription factor nf- *κ*B. *Free Radicals and Antioxidants*.

[83] Miles E. A., Calder P. C. (2021). Effects of citrus fruit juices and their bioactive components on inflammation and immunity: a narrative review. *Frontiers in Immunology*.

[84] Sandamali J. A. N., Hewawasam R. P., Jayatilaka K. A. P. W., Mudduwa L. K. B. (2021). Cinnamomum zeylanicum Blume (Ceylon cinnamon) bark extract attenuates doxorubicin induced cardiotoxicity in Wistar rats. *Saudi Pharmaceutical Journal*.

[85] Jayaprakasha G. K., Ohnishi-Kameyama M., Ono H., Yoshida M., Jaganmohan Rao L. (2006). Phenolic constituents in the fruits of cinnamomum zeylanicum and their antioxidant activity. *Journal of Agricultural and Food Chemistry*.

[86] Moselhy S. S., Ali H. K. (2009). Hepatoprotective effect of cinnamon extracts against carbon tetrachloride induced oxidative stress and liver injury in rats. *Biological Research*.

[87] Abdeen A., Abdelkader A., Abdo M. (2019). Protective effect of cinnamon against acetaminophen-mediated cellular damage and apoptosis in renal tissue. *Environmental Science and Pollution Research*.

[88] PubChem (2023). Cinnamaldehyde | C9H8O - PubChem. https://pubchem.ncbi.nlm.nih.gov/compound/Cinnamaldehyde.

[89] Liu Y., An T., Wan D., Yu B., Fan Y., Pei X. (2020). Targets and mechanism used by cinnamaldehyde, the main active ingredient in cinnamon, in the treatment of breast cancer. *Frontiers in Pharmacology*.

[90] Liao B. C., Hsieh C. W., Liu Y. C., Tzeng T. T., Sun Y. W., Wung B. S. (2008). Cinnamaldehyde inhibits the tumor necrosis factor-*α*-induced expression of cell adhesion molecules in endothelial cells by suppressing NF-*κ*B activation: effects upon I*κ*B and Nrf2. *Toxicology and Applied Pharmacology*.

[91] Cabello C. M., Bair W. B., Lamore S. D. (2009). The cinnamon-derived Michael acceptor cinnamic aldehyde impairs melanoma cell proliferation, invasiveness, and tumor growth. *Free Radical Biology and Medicine*.

[92] PubChem (2023). Cinnamic acid | C9H8O2 - PubChem. https://pubchem.ncbi.nlm.nih.gov/compound/Cinnamic-acid.

[93] Liu L., Hudgins W. R., Shack S., Yin M. Q., Samid D. (1995). Cinnamic acid: a natural product with potential use in cancer intervention. *International Journal of Cancer*.

[94] Niero E. L., Machado-Santelli G. M., de O., Machado-Santelli G M. (2013). Cinnamic acid induces apoptotic cell death and cytoskeleton disruption in human melanoma cells. *Journal of Experimental & Clinical Cancer Research*.

[95] Yen G. C., Chen Y. L., Sun F. M., Chiang Y. L., Lu S. H., Weng C. J. (2011). A comparative study on the effectiveness of cis- and trans-form of cinnamic acid treatments for inhibiting invasive activity of human lung adenocarcinoma cells. *European Journal of Pharmaceutical Sciences*.

[96] Tsai C. M., Sun F. M., Chen Y. L., Hsu C. L., Yen G. C., Weng C. J. (2013). Molecular mechanism depressing PMA-induced invasive behaviors in human lung adenocarcinoma cells by cis- and trans-cinnamic acid. *European Journal of Pharmaceutical Sciences*.

[97] Pandey K. B., Rizvi S. I. (2009). Plant polyphenols as dietary antioxidants in human health and disease. *Oxidative Medicine and Cellular Longevity*.

[98] Terra X., Palozza P., Fernandez-Larrea J. (2011). Procyanidin dimer B1 and trimer C1 impair inflammatory response signalling in human monocytes. *Free Radical Research*.

[99] Vetal S., Bodhankar S. L., Mohan V., Thakurdesai P. A. (2013). Anti-inflammatory and anti-arthritic activity of type-A procyanidine polyphenols from bark of Cinnamomum zeylanicum in rats. *Food Science and Human Wellness*.

[100] Ohnuma T., Matsumoto T., Itoi A. (2011). Enhanced sensitivity of A549 cells to the cytotoxic action of anticancer drugs via suppression of Nrf2 by procyanidins from Cinnamomi Cortex extract. *Biochemical and Biophysical Research Communications*.

[101] Krajka-Kuźniak V., Baer-Dubowska W. (2021). Modulation of Nrf2 and NF-*κ*B signaling pathways by naturally occurring compounds in relation to cancer prevention and therapy. Are combinations better than single compounds?. *International Journal of Molecular Sciences*.

[102] Moyle C. W., Cerezo A. B., Winterbone M. S. (2015). Potent inhibition of VEGFR‐2 activation by tight binding of green tea epigallocatechin gallate and apple procyanidins to VEGF: relevance to angiogenesis. *Molecular Nutrition & Food Research*.

[103] Masłowska K., Halik P. K., Tymecka D., Misicka A., Gniazdowska E. (2021). The role of VEGF receptors as molecular target in nuclear medicine for cancer diagnosis and combination therapy. *Cancers*.

